# Characterization and Comparison of Commercial Pumpkin (*Cucurbita moschata*) Seed Oils from Various Brands in China

**DOI:** 10.3390/foods15091602

**Published:** 2026-05-06

**Authors:** Yuan Gao, Xiaoyu Duan, Zhaoyu Chen, Li Zhou, Dongping He, Fenfen Lei

**Affiliations:** 1 Key Laboratory of Edible Oil Quality and Safety, State Administration for Market Regulation, School of Food Science and Engineering, Wuhan Polytechnic University, Wuhan 430023, China; g924704821@163.com (Y.G.); lrain_duan@163.com (X.D.); czyczy1019@126.com (Z.C.); zhouli@whpu.edu.cn (L.Z.); hedp123456@163.com (D.H.); 2 Grain and Oil Resources Comprehensive Exploitation and Engineering Technology Research Center of State Administration of Grain, Wuhan Polytechnic University, Wuhan 430023, China

**Keywords:** antioxidant capacity, fatty acid composition, physicochemical properties, pumpkin seed oil

## Abstract

Pumpkin seed oil (PSO) is becoming increasingly valued for its nutritional profile and minor bioactive constituents. Here, we surveyed PSOs marketed in China and hypothesized that differences in antioxidant constituents help explain variability in oxidative stability across brands. Sixteen commercial products labeled as pumpkin (*Cucurbita moschata Duchesne*) seed oils, including two imported brands (France), were analyzed for physicochemical quality indices, fatty acid composition, oxidative stability, and bioactive components (tocopherols, total sterols, and total phenolics expressed as gallic acid equivalents). Acid value, peroxide value, and iodine value ranged from 0.22 to 4.30 mg KOH/g, from 4.63 to 11.57 mEq O_2_/kg, and from 106.64 to 132.77 g I_2_/100 g, respectively, and all samples complied with applicable regulatory limits. Oleic and linoleic acids predominated (21.79–35.50% and 44.99–57.03% of total fatty acids, respectively), consistent with a highly unsaturated oil matrix. Total phenolics varied widely, reaching 2247.78 mg GAE/kg, while total tocopherols and total sterols ranged from 268.26 to 528.26 mg/kg and 733.64 to 1095.99 mg/kg, respectively. Oxidation induction times ranged from approximately 4 to over 10 h, and radical-scavenging activity differed markedly among samples. Correlation analysis and principal component analysis consistently identified δ-tocopherol, total sterols, and total phenolics as the variables most strongly associated with oxidative stability and antioxidant performance. Overall, these results provide a market-level snapshot of compositional variability in PSOs sold in China and highlight antioxidant-related markers that may support quality differentiation and process optimization.

## 1. Introduction

Pumpkin seeds, produced by several species within the genus Cucurbita (*Cucurbitaceae*), are widely consumed because of their nutrient density and associated health benefits [1]. Among these, *Cucurbita moschata* is widely grown across China, which leads global pumpkin production with yields exceeding 7.5 million tons annually. The seeds contain approximately 35–50% oil by dry weight, and in some cultivars, the oil content can reach up to 64.4%, making them a promising source of edible oil [2]. Pumpkin seed oil (PSO) has also been used in traditional practice, particularly for gastrointestinal complaints and urogenital symptoms, and contemporary studies have reported lipid-modulating effects and potential benefits for benign prostatic hyperplasia [3,4,5]. These effects have been attributed to PSO’s fatty acid profile, together with minor constituents such as phytosterols, tocopherols, and phenolic compounds that contribute to antioxidant and biological activity [6]. Beyond its nutritional value, PSO has been reported to exhibit antimicrobial, antioxidant, anti-inflammatory, and anticancer activities in experimental models [6,7]. In parallel, formulation strategies such as nanoemulsification and ozonation have been explored to improve dispersibility, stability, and functional performance of seed oils, including PSO [8,9]. However, the composition and functionality of PSO are not intrinsic constants. They are shaped by genotype, climate, soil conditions, and agronomic practices, which collectively influence seed lipid accumulation and fatty acid distribution [10,11]. Processing conditions further modulate sensory quality and the retention of thermolabile bioactives. Cold pressing is widely used because it can preserve natural pigments and minor compounds [6], whereas supercritical CO_2_ extraction is often applied to enhance extraction efficiency while avoiding organic solvents and, in some cases, improving preservation of selected bioactive compounds [12]. Packaging and storage conditions can also influence oxidative deterioration, particularly for oils rich in polyunsaturated fatty acids. Importantly, “pumpkin seed oil” is a market term rather than a botanical guarantee. Globally, PSO is commonly produced from hull-less *Cucurbita pepo* L. var. *styriaca*, while oils from *C. maxima Duchesne* and *C. moschata Duchesne* have also been reported, each with distinct compositional signatures [13]. European studies have documented substantial variation in fatty acids across species and growing regions [10]. In contrast, comprehensive, market-oriented data for PSO products sold in China remain limited, despite the diversity of cultivars, regional supply chains, and processing practices. In addition, commercial product labels often provide incomplete information on seed pretreatment (for example, roasting), storage duration, and packaging, all of which can affect indices such as acid value (AV), peroxide value (PV), phenolic measurements, and oxidative stability. As a result, a systematic comparison across brands is needed to clarify the practical ranges of quality and to identify compositional markers that best explain stability differences.

Accordingly, this study characterized and compared the quality attributes of 16 commercial PSO products marketed in China. Physicochemical indices (AV, PV, and iodine value), fatty acid profiles, oxidative stability (Rancimat induction time), radical-scavenging activity, and key bioactive constituents (tocopherols, total sterols, and total phenolics) were determined. Correlation analysis and principal component analysis (PCA) were further employed to investigate whether variability in oxidative stability among commercial PSOs is more strongly associated with antioxidant-related minor constituents than with bulk fatty acid composition. By integrating compositional data with stability and multivariate statistics, the work provides a market-level assessment that can support quality control, product differentiation, and processing optimization for PSO production and commercialization.

## 2. Materials and Methods

### 2.1. Materials and Chemicals

Sixteen commercial pumpkin seed oil (PSO) products were purchased from supermarkets and major e-commerce platforms in China. According to label declarations, the oils were derived from seeds of *Cucurbita moschata Duchesne*. Product origin, declared processing method, and packaging type are summarized in Table 1. Information on production date, shelf life, seed pretreatment (for example, roasting), and storage conditions was not consistently disclosed across brands; therefore, all analyses were performed on products as received at the time of purchase. Wijs reagent, gallic acid standard, potassium hydroxide (KOH) titrant, 2,2-diphenyl-1-picrylhydrazyl (DPPH), 2,2′-azinobis (3-ethylbenzothiazoline-6-sulfonic acid) (ABTS), and α-, γ-, and δ-tocopherol standards were obtained from Sigma-Aldrich (Beijing, China). Reagents and standards were protected from light and stored in amber containers at 4 °C until use. Unless otherwise specified, all other solvents and reagents used in this study were of analytical grade and purchased from Aladdin (Shanghai, China).

### 2.2. Determination of Physical and Chemical Properties

#### 2.2.1. Determination of Acid Value

Approximately 10 g of each oil sample was accurately weighed into a 250 mL Erlenmeyer flask. A mixed solvent of diethyl ether and isopropanol (50.0 mL) was added, followed by phenolphthalein indicator (four drops). The mixture was shaken until the oil was fully dissolved and then titrated with standardized KOH solution. The endpoint was defined as a faint pink color persisting for at least 15 s [14,15]. Acid value (AV) was calculated as

(1) $$\mathrm{AV}\;(\mathrm{mg}\;(\mathrm{KOH})/g)=\frac{(V-{\;V}_{0})\;\times \;C\;\times \;56.11}{m}$$

where V (mL) is the volume of KOH used for the sample, V_0_ (mL) is the volume used for the blank, C (mol/L) is the molar concentration of KOH, and m (g) is the sample mass.

#### 2.2.2. Determination of Iodine Value

Iodine value (IV) was determined following the AOAC Official Method 993.20 [16]. The oil sample was dissolved in a cyclohexane and glacial acetic acid mixture (10 mL + 10 mL, 1:1, *v*/*v*). Wijs reagent (25 mL) was added, and the mixture was kept in the dark for 1 h. A 10% potassium iodide solution (20 mL) was then added, followed immediately by distilled water (150 mL). The released iodine was titrated with standardized sodium thiosulfate solution (0.1 N) with continuous swirling. When the solution became pale yellow, starch indicator was added, and titration was continued until the blue color disappeared. IV was calculated as

(2) $$\mathrm{IV}=\frac{(V_{0}-V)\;\times \;C\;\times \;12.69}{m}$$

where V (mL) is the volume of sodium thiosulfate used for the sample, V_0_ (mL) is the volume used for the blank, C (mol/L) is the concentration of sodium thiosulfate, and m (g) is the sample mass [17,18].

#### 2.2.3. Determination of Peroxide Value

Peroxide value (PV) was measured by iodometric titration. Oil (2.0 g) was transferred into a 250 mL conical flask and dissolved in a trichloromethane and glacial acetic acid mixture (30 mL). Saturated potassium iodide solution (1.00 mL) was added, the flask was sealed and gently shaken for 30 s, and the mixture was kept in the dark for 3 min. Distilled water (100 mL) was added, and the liberated iodine was titrated immediately with standardized sodium thiosulfate solution. Near the endpoint, starch indicator (1 mL) was added, and titration was continued until the blue color disappeared. PV was calculated as shown in (3):

(3) $$PV=\frac{\left(V-V_{0}\right)\times C\times 1000}{m}$$

where V (mL) is the volume of sodium thiosulfate used for the sample, V_0_ (mL) is the volume used for the blank, C (mol/L) is the concentration of sodium thiosulfate, and m (g) is the sample mass [19].

### 2.3. Fatty Acid Composition Determination

Fatty acid methyl esters (FAMEs) were prepared by alkaline transesterification as previously described [20]. Briefly, 50 mg of oil was mixed with 2 mL of n-hexane and 0.2 mL of 2 mol/L methanolic potassium hydroxide. The mixture was vortexed for 1 min and allowed to phase-separate, after which the upper n-hexane layer containing FAMEs was collected for chromatographic analysis. FAMEs were analyzed by gas chromatography with flame ionization detection (GC-FID) using an Agilent 7890B system (Agilent Technologies, Santa Clara, CA, USA) equipped with a DB-WAX capillary column (30 m × 0.25 mm × 0.25 μm). A 1 μL aliquot of the prepared fatty acid methyl ester (FAME) extract was injected into the chromatograph. The inlet temperature was maintained at 250 °C. High-purity helium served as the carrier gas, operated under a split injection mode with a split ratio of 30:1. The column flow rate was maintained at 1 mL/min throughout the analysis. The oven temperature program was optimized to achieve efficient separation of fatty acid components. The temperature was initially set at 70 °C and held for 3 min. It was then increased to 180 °C at a rate of 10 °C/min, followed by a slower ramp to 200 °C at 2 °C/min with a 1-min hold. Finally, the temperature was raised to 220 °C at 3 °C/min and held for 15 min. Fatty acid components were identified by comparing their retention times with those of known FAME standards. The relative percentage of each fatty acid was calculated based on peak area normalization.

### 2.4. Bioactive Compounds Determination

#### 2.4.1. Determination of Total Phenol Content

Total phenolic content was measured after methanolic extraction of oil. Briefly, 1.0 g of PSO was mixed with 5.0 mL of methanol, thoroughly vortexed, and centrifuged at 6000 rpm and 4 °C for 6 min. The supernatant was transferred to a 25 mL volumetric flask. Extraction was repeated three times, and pooled extracts were brought to volume with methanol. An aliquot (0.2 mL) was measured at 750 nm using a UV–Vis spectrophotometer (UV-2450, Shimadzu, Kyoto, Japan). Quantification was performed using an external calibration curve of gallic acid, and results were expressed as mg gallic acid equivalents (GAE) per kg oil. Because spectrophotometric total phenolic assays are based on redox chemistry and can respond to non-phenolic reducing compounds, particularly in oils produced from thermally treated seeds, the values obtained were interpreted as comparative indices rather than compound-specific concentrations.

#### 2.4.2. Tocopherol Determination

Tocopherol isomers (α-, γ-, and δ-tocopherol) were quantified by high-performance liquid chromatography with UV detection (HPLC-UV) using an UltiMate 3000 system (Thermo Scientific, Shanghai, China) equipped with a Venusil XBP C18 column (5 μm, 4.6 mm × 250 mm) [21]. Methanol was used as the mobile phase under isocratic elution at 1.0 mL/min. The column temperature was maintained at 25 °C, and the injection volume was 2 μL. Detection was performed at 294 nm. Peaks were identified by retention time matching to authentic standards and quantified using external calibration. The calibration relationship was y = 78.801x − 0.0463 (R^2^ = 0.9994), where y is detector response and x is tocopherol concentration. Full analytical validation parameters, including limit of detection, limit of quantification, recovery, and repeatability/reproducibility, were not comprehensively established for the present dataset; therefore, the reported tocopherol values should be interpreted as quantitative estimates obtained under the stated HPLC-UV conditions.

#### 2.4.3. Determination of Sterol Content

Total sterols were detected using a sulfate–phosphate–ferric method with minor modifications [22]. Briefly, 2.0 g of each PSO sample was saponified with 20 mL ethanolic potassium hydroxide (1 mol/L) in a round-bottom flask at 90 °C for 30 min. After cooling, the saponified mixture was transferred to a separatory funnel using diethyl ether and distilled water, and then subjected to phase separation. The aqueous layer was discarded, and the organic phase was washed repeatedly with water until neutral. The organic layer was concentrated using rotary evaporation, and the residue was dissolved in anhydrous ethanol and adjusted to 25 mL. A 2 mL aliquot of this solution was transferred to a test tube, followed by the addition of 2 mL of anhydrous ethanol and 2 mL of iron phosphorothioate chromogenic reagent. The mixture was allowed to react at room temperature for 15 min to develop color. Absorbance was measured at 442 nm using a UV-visible spectrophotometer. Results were expressed in milligrams of total sterols per 100 g of oil, calculated against a standard curve for soy sterol equivalents: y = 3.7386x - 0.0373 (R^2^ = 0.9997). Given the non-specific nature of colorimetric sterol assays and the compositional differences between soy sterols and the Δ7-sterols typical of pumpkin seed oil, the results were treated as approximate estimates suitable for comparison among samples rather than absolute sterol speciation.

### 2.5. Measurement of Oxidative Stability and Free Radical Scavenging Capacity

#### 2.5.1. Oxidation Stability

Oxidative stability was evaluated using a Rancimat instrument (Model 892, Metrohm, Herisau, Switzerland). Oil samples were subjected to accelerated oxidation at 106.6 °C under a constant airflow of 15 L/h. These conditions were selected to provide measurable induction times for highly unsaturated seed oils and to enable comparisons among commercial products. The induction time was recorded as the point at which a rapid increase in conductivity was observed in the measuring vessel containing distilled water that captured volatile oxidation products, and it was used as an operational indicator of oxidative stability.

#### 2.5.2. ABTS Cationic Radical and DPPH Radical Scavenging Ability

Radical scavenging activity was assessed using DPPH and ABTS assays based on published protocols with minor modifications [23,24]. Methanolic extracts were prepared as described in Section 2.4.1. For the DPPH assay, 100 μL of extract was mixed with 3.0 mL of 0.12 mmol/L DPPH solution in methanol. The mixture was incubated in the dark at room temperature for 30 min, and absorbance was measured at 517 nm against the appropriate blank. For the ABTS assay, ABTS radical cations were generated by reacting 7 mmol/L ABTS with 2.45 mmol/L potassium persulfate in the dark for 12–16 h. The ABTS working solution was diluted to an absorbance of 0.70 ± 0.02 at 734 nm. Then, 100 μL of extract was added to 1.0 mL of the ABTS working solution, and absorbance was recorded at 734 nm after 6 min. For both assays, results were expressed as percentage inhibition, calculated as

(4) $$Inhibition\;(\%)=\left[1-(\frac{A_{sample}-A_{blank}}{A_{control}})\right]\times 100$$

where *A_control_* is the absorbance of the radical solution without extract, *A_sample_* is the absorbance after reaction with extract, and *A_blank_* accounts for background absorbance of the extract in the corresponding solvent system.

### 2.6. Colorimetric Analysis

Color characteristics were assessed using a portable colorimeter (CR-400, Konica Minolta, Tokyo, Japan) equipped with a D65 illuminant and 10° standard observer. Oil samples were placed in 10 mm quartz cuvettes, and L* (lightness), a* (red-green), and b* (yellow-blue) values were recorded. All measurements were conducted in triplicate, and mean values were used for comparison.

### 2.7. Statistical Analysis

All analytical measurements were performed in triplicate for each oil sample, and data are presented as mean ± standard deviation (*n* = 3). Statistical analyses were conducted using SPSS 19.0 (IBM Corp., Armonk, NY, USA). Principal component analysis (PCA), hierarchical cluster analysis, and data visualization were performed using OriginPro 2025 (OriginLab Corporation, Northampton, MA, USA). Differences among samples were evaluated using one-way analysis of variance (ANOVA). When ANOVA indicated significance, post hoc comparisons were performed using the least significant difference (LSD) test and Duncan’s multiple range test as appropriate. Statistical significance was set at *p* < 0.05. Associations between compositional indices and antioxidant-related measurements were evaluated using correlation analysis (two-tailed), and correlation coefficients were reported to describe the strength and direction of relationships. Multivariate analyses were performed to support pattern recognition across commercial products. Before principal component analysis (PCA), variables were standardized to z-scores to minimize scaling effects. Principal components were retained based on eigenvalues greater than 1 in combination with inspection of the scree plot. Hierarchical cluster analysis was conducted to visualize sample grouping based on shared characteristics, and clustering results were used to support the interpretation of PCA-derived patterns.

## 3. Results and Discussion

### 3.1. Physicochemical Properties and Color of PSOs

**Figure 1:**
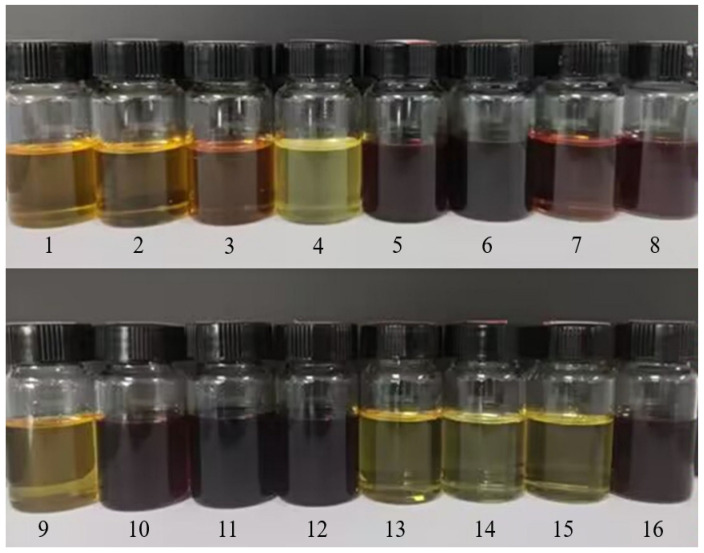
Appearance of different pumpkin seed oils.

The physicochemical characteristics of the 16 PSO samples are summarized in Table 2. The AV ranged from 0.22 to 4.30 mg KOH/g oil, the IV from 106.64 to 132.77 g I_2_/100 g oil, and the PV from 4.63 to 11.57 mEq O_2_/kg oil. All values fell within the applicable limits of Chinese standards for edible vegetable oils and were consistent with the quality criteria described in the Codex Alimentarius Standard for Named Vegetable Oils (CXS 210-1999). AV reflects the level of free fatty acids and is therefore a practical indicator of hydrolytic deterioration. Samples 4, 14, and 15 exhibited particularly low AVs (≤0.4 mg KOH/g), suggesting limited hydrolysis and, by extension, good preservation of triacylglycerols. This range is broadly consistent with published AVs reported for PSO (approximately 1.56 ± 0.04 mg KOH/g) [25]. IV reflects the degree of unsaturation and represents grams of iodine absorbed per 100 g of oil. Most samples showed IVs above 120 g I_2_/100 g, except for samples 1–5 and 7, indicating substantial differences in unsaturation among commercial products. In line with the classification proposed by Lazos [26], all samples can be considered highly unsaturated oils (IV > 100 g I_2_/100 g), which is nutritionally favorable but may increase oxidative susceptibility. PV serves as an indicator of primary oxidation products (hydroperoxides) and is particularly sensitive to oxygen exposure, light, trace pro-oxidants, and storage duration. Although all PVs remained below regulatory thresholds, the observed spread (4.63–11.57 mEq O_2_/kg) indicates meaningful differences in early-stage oxidation among brands. The highest PVs were observed in samples 10 and 11 (≥10.8 mEq O_2_/kg), whereas sample 3 showed the lowest PV (4.63 mEq O_2_/kg). Given that commercial labels did not consistently report production date, pretreatment conditions (for example, roasting), or storage history, PV variation is most plausibly interpreted as an integrated outcome of raw material quality, processing control, packaging, and market circulation time rather than a single controllable factor. This variability is relevant because elevated PV, even within acceptable limits, can accelerate downstream formation of secondary oxidation compounds and adversely affect sensory stability. Color and visual appearance differed markedly among samples (Figure 1; Table 2). Significant differences were observed in CIE Lab* parameters (*p* < 0.05), indicating variation in lightness (L*), red-green tone (a*), and yellow-blue tone (b*). L* values were highest in samples 1, 14, and 15, consistent with a clearer, lighter appearance, whereas sample 11 exhibited the lowest L*, corresponding to a visibly darker oil. Several samples showed a* values near zero (for example, 1–4, 9, 13–15), indicating minimal red or green bias, while other samples had positive a* values, reflecting a redder hue. All oils displayed relatively high b* values, consistent with a strong yellow component. The darker appearance of some samples (for example, 5, 6, 8, 11, and 12) may reflect higher concentrations of pigments and co-extracted minor compounds, which can be influenced by cultivar, seed maturity, thermal pretreatment, and filtration or refining steps such as dewaxing or decolorization. A visual transition from yellow to green under thin-film illumination was also noted, which is consistent with light-dependent pigment behavior attributed to chlorophyll-related compounds, as previously reported by Kreft et al. [27]. Importantly, color should be interpreted cautiously as a quality indicator because pigments may covary with bioactives, yet they can also promote photo-oxidation depending on their composition and the packaging light barrier.

### 3.2. Fatty Acid Composition of PSOs

Fatty acid profiles are presented in Table 3. All PSO samples contained palmitic (C16:0), stearic (C18:0), oleic (C18:1), linoleic (C18:2), and linolenic (C18:3) acids, with oleic and linoleic acids predominating. Minor fatty acids such as lauric (C12:0) and tricosanoic (C23:0) acids were detected only in a few samples, reflecting variability in composition likely influenced by seed genotype and regional climate [10]. These findings are in close agreement with global PSO data reported by Potocnik et al. [28], who analyzed 38 PSO samples from different geographical origins and observed oleic acid contents ranging from 26.8% to 43.6% and linoleic acid contents from 37.2% to 54.9%. In the present study, linoleic acid generally exceeded oleic acid, and other fatty acids contributed only modestly, which is in agreement with previously reported PSO patterns [29]. Total unsaturated fatty acid content ranged from 74.36% to 91.67%, with PUFAs exceeding monounsaturated fatty acids (MUFAs). This profile resembles that of sesame and peanut oils. Oleic acid (C18:1), a major MUFA, is known to reduce low-density lipoprotein (LDL) cholesterol, while linoleic acid (C18:2), a PUFA, is linked to cardiovascular and developmental health benefits. This compositional signature supports the nutritional positioning of PSO as a PUFA-rich seed oil. At the same time, high PUFA content can reduce thermal and oxidative robustness, particularly under high-temperature cooking. Consequently, these oils may be better suited to low-heat culinary applications or as blending components with more oxidation-resistant oils when thermal exposure is expected [30]. This compositional context is important for interpreting the stability results reported below, because oxidative performance in highly unsaturated oils is often determined not only by fatty acid distribution but also by the abundance and reactivity of minor antioxidants.

### 3.3. Bioactive Compounds of PSOs

#### 3.3.1. Total Phenolic Content of PSOs

**Figure 2:**
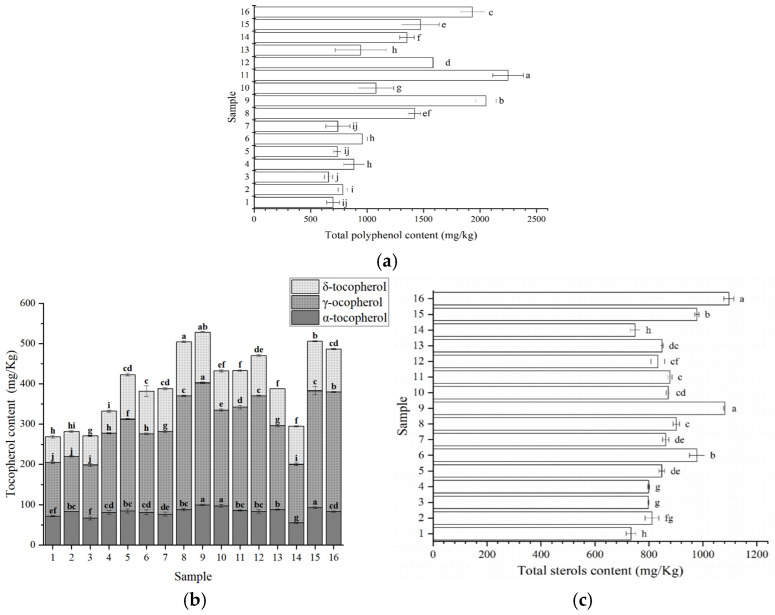
Total phenolic content (**a**), tocopherol composition (**b**) and total sterol content (**c**) of pumpkin seed oil. Different lowercase letters indicate statistically significant differences among samples (*p* < 0.05).

The health-promoting properties of PSO are influenced not only by its fatty acid matrix but also by minor bioactive constituents. Among these, phenolic-related compounds contribute to antioxidant behavior and may influence shelf-life performance [31]. As shown in Figure 2a, total phenolic content differed significantly among the PSO samples (*p* < 0.05), indicating pronounced heterogeneity across the market. Sample 13 exhibited the highest total phenolic content (2247.78 mg GAE/kg), while samples 8, 9, 10, 11, 14, 15, and 16 also displayed relatively high values exceeding 1000 mg GAE/kg. In contrast, the remaining samples contained substantially lower concentrations. Such variability may reflect differences in cultivar, agronomic conditions, seed maturity, and pre-extraction treatments, as well as processing and storage practices [30]. However, interpretation requires methodological caution. Total phenolic content was determined using the Folin–Ciocalteu assay, which is based on redox reactions and is not specific to phenolic compounds. The response may include other reducing substances, including Maillard reaction products formed during roasting or thermal pretreatment, as well as other redox-active constituents that partition into the methanolic extract. Therefore, the reported values should be interpreted as comparative indices of extractable reducing capacity rather than direct measurements of phenolic speciation. Quantitative comparisons with other oils are only meaningful when extraction procedures and assay conditions are closely matched. Although the values observed here exceed those reported for rice bran oil, soybean oil, and several other vegetable oils, such comparisons should be treated cautiously and contextualized by methodological differences [32,33]. Future work using compound-resolved approaches (for example, chromatographic profiling) would be required to confirm the specific phenolic composition and to identify the compounds most responsible for oxidative protection in PSO.

#### 3.3.2. Tocopherol Composition of PSO

Tocopherols are lipid-soluble antioxidants that can terminate free-radical chain reactions by donating hydrogen atoms to lipid peroxyl radicals, thereby slowing the propagation stage of lipid oxidation. They also represent the major vitamin E activity in most edible oils. As shown in Figure 2b, three types of tocopherols were detected in the PSO samples: α-tocopherol, γ-tocopherol, and δ-tocopherol. Among these, γ-tocopherol was the dominant isomer, accounting for approximately 60% of total tocopherols, which is consistent with reports that γ-tocopherol is a major tocopherol form in many seed oils and can contribute substantially to oxidative protection in PUFA-rich matrices. Beyond antioxidant activity, γ-tocopherol has been associated with cardiometabolic protective effects in experimental and epidemiological contexts [34]. Total tocopherol concentrations differed significantly among samples (268.26–528.26 mg/kg). The highest levels were observed in samples 8, 9, 15, and 16, whereas samples 1, 2, 3, and 14 exhibited the lowest values. Because the products were collected from the market and label information did not consistently specify seed pretreatment, production date, or storage duration, the observed dispersion is best interpreted as the combined outcome of cultivar background, growing environment, and processing and storage conditions rather than a single determinant. Environmental regulation of tocopherol biosynthesis under abiotic stress has been reported, and factors such as water availability may modulate tocopherol accumulation in oil-bearing tissues [35]. The total tocopherol levels measured here were lower than those reported for selected cold-pressed PSOs in other markets, although the relative isomer distribution was broadly similar [36]. Internationally, Styrian PSOs have been reported to span a wide range (approximately 255–1000 mg/kg), indicating that the values observed in this study fall within the lower to mid portion of the reported range [37]. It should also be noted that PSO can contain additional tocol derivatives, including γ-tocomonoenol and α-tocomonoenol, which were not quantified in the present analysis but have been detected previously, particularly in oils derived from roasted seeds. These compounds may contribute to oxidative behavior and should be included in future work using broader chromatographic profiling.

#### 3.3.3. Total Sterols of PSO

Sterols are naturally occurring compounds in vegetable oils known for their antioxidant properties and cholesterol-lowering effects. In combination with the high linoleic acid content of PSO, sterols may offer additional benefits in managing lipid-related conditions such as atherosclerosis [38]. As shown in Figure 2c, sterol content ranged from 733.64 to 1095.99 mg/kg, which is comparable to reported levels in several widely consumed vegetable oils [39]. Samples 9 and 16 exhibited the highest sterol estimates, while samples 1, 3, 4, and 14 showed the lowest values. Interpretation of sterol results requires methodological caution. Total sterols were estimated using a colorimetric method and expressed as soy sterol equivalents, whereas PSO is known to contain substantial proportions of Δ7-sterols. Because the chromogenic response can differ across sterol structures, the reported values should be regarded as approximate estimates suitable for relative comparison among samples rather than absolute sterol concentrations or compositional speciation. The lower sterol estimates were observed in several cold-pressed products, but the overall pattern suggests that the extraction method alone is unlikely to explain the variability. Other factors such as cultivar background, post-harvest handling, processing intensity, and storage conditions may contribute to differences in sterol recovery and retention [37]. Accordingly, any association between sterol level and antioxidant performance should be interpreted in the context of these analytical and supply-chain uncertainties.

### 3.4. Oxidation Stability of PSO

**Figure 3:**
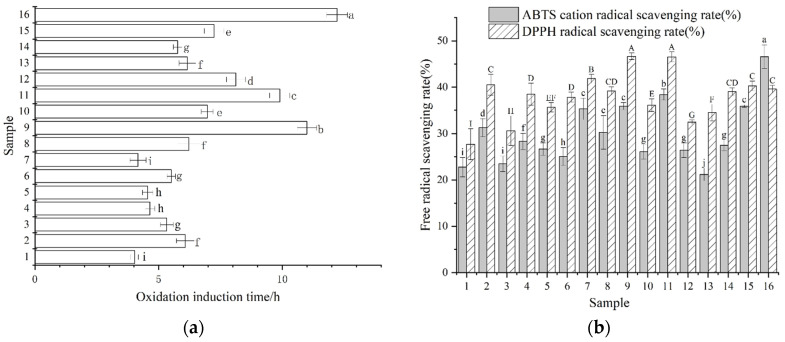
Oxidation induction time (**a**) and radical scavenging activity (**b**) of pumpkin seed oil. Different lowercase letters in panel (**a**) indicate significant differences among samples in oxidation induction time (*p* < 0.05). In panel (**b**), different uppercase letters indicate significant differences in ABTS cation radical scavenging rate, whereas different lowercase letters indicate significant differences in DPPH radical scavenging rate (*p* < 0.05).

Oxidative stability was evaluated by Rancimat induction time (Figure 3a). Induction times differed significantly among the 16 commercial products, indicating substantial variability in resistance to accelerated oxidation. Samples 9 and 16 demonstrated the highest oxidative stability, with induction times exceeding 10 h, whereas samples 1, 4, 5, and 7 exhibited induction times below 5 h. This range is consistent with values reported for PSO in prior studies (approximately 4.5–12.93 h), supporting that the observed dispersion is plausible for oils differing in composition and processing history [40]. Studies have further shown that thermal pretreatments such as roasting or microwave exposure can increase induction time, partly through the formation of antioxidative Maillard reaction products, including 5-hydroxymethylfurfural, which may contribute to radical scavenging and delayed peroxide accumulation [41]. Because pretreatment conditions were not consistently declared for the commercial samples analyzed here, the contribution of roasting-derived products cannot be separated from native antioxidants. The variability in induction time likely reflects an interplay among fatty acid unsaturation, antioxidant composition, and extrinsic factors such as oxygen exposure, light transmission through packaging, and storage duration before purchase. Oxidation is relevant not only because it diminishes sensory and nutritional quality, but also because it promotes the accumulation of secondary oxidation products during storage. For practical use, PSO is therefore best stored in cool, dark conditions after opening and consumed within a reasonable period, particularly for oils packaged in light-permeable containers. To complement induction time, radical scavenging capacity was assessed using DPPH and ABTS assays (Figure 3b) [42]. The DPPH scavenging rate ranged from 35.17% to 71.98%, and the ABTS scavenging rate ranged from 27.70% to 46.68%. Samples 7, 9, 11, and 16 exhibited the highest antioxidant activity in both assays, while samples 1, 3, and 13 showed relatively lower values. These results suggest that samples with higher oxidative stability also possessed greater radical scavenging capacities, reinforcing the link between antioxidant content and oil stability. Notably, samples 9 and 16 combined high sterol estimates and higher tocopherol levels with prolonged induction times and relatively strong scavenging activity. Mechanistically, tocopherols can interrupt lipid peroxidation via hydrogen donation, and δ-tocopherol is often considered particularly effective in suppressing oxidation in oil systems because of its reactivity profile. Sterols may also modulate oxidation by interacting with radical intermediates and influencing the stability of oxidation products, although the magnitude of this contribution depends on sterol structure and the broader minor-compound matrix.

Finally, the limitations of these antioxidant metrics should be acknowledged. DPPH and ABTS assays are performed in solvent extracts and can be influenced by non-phenolic reducing compounds, especially in oils produced from thermally treated seeds. As such, they should be interpreted primarily as comparative indicators of extractable redox activity rather than direct measures of antioxidant efficacy within the intact oil phase. Similarly, Rancimat induction time reflects susceptibility to oxidation under accelerated high-temperature conditions, and it should be treated as a standardized ranking tool rather than a direct predictor of shelf life under ambient storage.

### 3.5. Principal Component Analysis and Correlation Analysis

To examine relationships among key quality indicators, correlation analysis was conducted between antioxidant-related constituents (total phenolics, total sterols, and individual tocopherol isomers), selected fatty acids, oxidative stability (Rancimat induction time, reported here as the oxidative stability index, OSI), and radical scavenging activity (Table 4). PCA was used as a complementary multivariate tool to summarize variance across samples and to support pattern recognition. It should be noted that PCA explains statistical variance and does not establish mechanistic causality. Accordingly, variable loadings should be interpreted as indicators of their contribution to sample discrimination rather than as proof of exclusive determinants of oxidative stability. Total phenolics showed a significant positive association with both OSI and DPPH radical scavenging activity, which is consistent with prior observations that phenolic-related constituents contribute to oxidative protection in lipid systems [43]. Total sterols also displayed positive associations with antioxidant indices, supporting the view that sterol-related components can contribute to improved oxidative resistance, although the strength of this relationship should be interpreted cautiously, given that sterols were estimated by a colorimetric method. In contrast, α-tocopherol and γ-tocopherol were not significantly correlated with OSI or radical scavenging activity in this dataset. This pattern agrees with reports that tocopherol isomers may differ in antioxidant effectiveness depending on oil matrix composition and assay conditions, and that α- and γ-tocopherol do not always track directly with stability outcomes in PSO [44].

**Figure 4:**
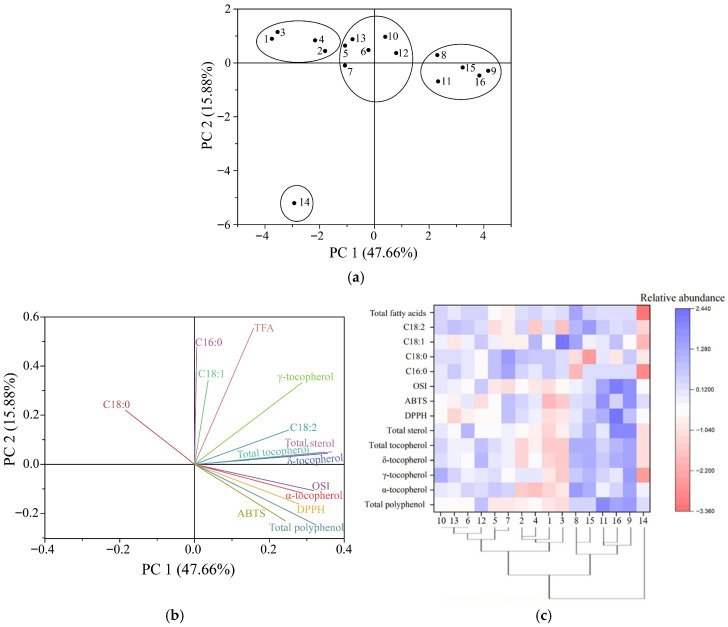
PCA of 14 major components of PSO: (**a**) Score plot of PCA of 16 samples; (**b**) Score plot of PCA of the main indicators; (**c**) Cluster heat map analysis.

Among tocopherol isomers, δ-tocopherol was the only form that showed significant positive associations with both oxidative stability and extractable radical scavenging activity. Specifically, δ-tocopherol correlated with OSI (r = 0.613, *p* < 0.05) and with DPPH inhibition (r = 0.690, *p* < 0.01). In contrast, neither α-tocopherol nor γ-tocopherol was significantly associated with OSI or radical scavenging indices. This pattern is consistent with the observations of Murkovic and Pfannhauser [44], who reported that δ-tocopherol can contribute more effectively to stability in PSO, potentially because of differences in structure and reactivity compared with other tocopherol isomers. Importantly, the major unsaturated fatty acids (oleic and linoleic acids) did not show significant correlations with OSI. This finding suggests that, within the range of fatty acid variability observed across the commercial products, oxidation resistance may be more strongly modulated by minor constituents than by bulk fatty acid composition. Alternatively, it may indicate that unmeasured factors that influence peroxidation kinetics, such as chlorophyll-related pigments, trace metals, or other pro-oxidants and antioxidants, contributed to the oxidation response but were not captured in the present dataset [45]. These interpretations are also compatible with the methodological context, because OSI reflects accelerated oxidation behavior under standardized conditions, in which minor antioxidants can exert a disproportionate influence on induction time. To further evaluate multivariate relationships and sample grouping, PCA was applied to 14 indicators, including total phenolics, sterols, α-, γ-, and δ-tocopherols, four major fatty acids, and antioxidant markers. PCA reduces dimensionality by summarizing correlated variables into orthogonal principal components that explain the variance across samples. In the loading plot, variables with vectors oriented at angles <90° are interpreted as positively related, whereas those separated by angles >90° are interpreted as negatively related [46]. Principal components were retained based on eigenvalues >1 in combination with inspection of the scree plot. The first two components (PC1 and PC2) explained 63.54% of the total variance, with PC1 accounting for 47.66% and PC2 contributing 15.88%. PC3 accounted for only a minor proportion of the total variance and did not materially affect the clustering pattern; therefore, subsequent interpretation focused on PC1 and PC2. The score plot (Figure 4a) separated the 16 PSO samples into four clusters, and sample 14 formed a distinct group, indicating a markedly different compositional profile. The loading plot (Figure 4b) indicated that total phenolics, total sterols, total tocopherols, and δ-tocopherol contributed most strongly to variance across products. These variables were aligned with the direction of higher stability and antioxidant-related performance, suggesting that they are key descriptors of quality differences among market samples rather than definitive causal determinants. Cluster patterns were broadly consistent with compositional trends. Samples 1–4 were grouped and characterized by relatively higher C18:0 and comparatively lower concentrations of antioxidant-related constituents. Samples 5, 6, 7, 10, 12, and 13 formed a second group with intermediate levels of C16:0, C18:0, and tocopherols. Samples 8, 9, 11, 15, and 16 were positioned farther from the origin, consistent with higher levels of tocopherols and sterols and, in several cases, higher unsaturated fatty acid proportions. Sample 14 remained separated from all other products, consistent with its distinctive tocopherol pattern, particularly elevated γ-tocopherol, together with an overall profile that diverged from the dominant trends observed for the remaining oils.

Overall, the multivariate results indicate that differences in PSO quality traits across commercial products are more closely aligned with antioxidant-related constituents than with any single fatty acid. Higher values of linoleic acid, tocopherols, and sterols were generally observed in samples 8, 9, 11, 15, and 16, and these samples tended to show stronger oxidative stability and antioxidant-related responses. However, a high abundance of one component should not be interpreted as sufficient evidence of superior overall quality. For example, sample 14 exhibited relatively high γ-tocopherol but did not show a similarly elevated profile across other stability-associated indicators, underscoring that quality assessment should be based on a compositional pattern rather than a single marker. A clustered heat map (Figure 4c) was used to visualize the relative distribution of indicators across samples and to support the PCA-based grouping. The clustering pattern was broadly consistent with the PCA score plot. Samples 9, 11, and 16 formed a distinct cluster characterized by higher relative levels of antioxidant-related constituents and higher oxidative stability indices. A second cluster (samples 5, 6, 7, 10, 12, and 13) exhibited intermediate profiles across most variables. Samples 1–4 were grouped and characterized by comparatively lower levels of antioxidant-related indicators and lower antioxidant activity. Sample 14 again appeared as an isolated branch, consistent with its divergent compositional pattern relative to the remaining products. Taken together, these analyses support a multifactorial view of PSO quality. In this dataset, δ-tocopherol, total sterols, and total phenolics emerged as the variables most consistently associated with oxidative stability and radical scavenging indices, and they also contributed strongly to sample separation in PCA. Because sterols were quantified using a colorimetric method (reported as equivalents) and total phenolics were measured by a redox-based assay, these variables should be interpreted as comparative markers rather than compound-resolved measures. Even with these analytical constraints, the convergence between correlation analysis and multivariate clustering suggests that antioxidant-related constituents provide practical descriptors for differentiating PSO products marketed in China. From an application perspective, preservation of δ-tocopherol and sterol-related fractions should be considered during processing and storage, and improved label transparency regarding processing and packaging could facilitate more informed quality selection.

## 4. Conclusions

This study demonstrated substantial variability in the compositional profiles of pumpkin seed oil (PSO) products marketed in China. Across the 16 commercial samples analyzed, key quality indices, including AV, PV, and IV, remained within applicable standards, indicating that the products met baseline regulatory requirements. The oils were characterized by a high degree of unsaturation, with an average unsaturated fatty acid proportion exceeding 85% and PUFAs generally exceeding MUFAs. This profile differs from that of olive oil and is more comparable to other PUFA-rich seed oils such as flaxseed, sunflower, and soybean oils. In addition to bulk fatty acids, the samples contained measurable levels of bioactive constituents, including tocopherols, sterols, and extractable phenolic-related reducing compounds, but large differences were observed among brands. Despite compliance with basic quality limits, oxidative stability varied markedly. More than half of the samples exhibited relatively short Rancimat induction times, suggesting reduced resistance to accelerated oxidation and potential implications for storage robustness after opening. Differences in tocopherols and sterols were also evident when compared with ranges reported in other markets, indicating that commercial PSO composition is shaped by multiple factors, including cultivar background, growing environment, processing intensity, and downstream storage and distribution conditions. Because production date, seed pretreatment (for example, roasting), and storage history were not uniformly disclosed on labels, variability across products is most appropriately interpreted as a combined market-chain effect rather than being attributed to a single controllable variable. Statistical analyses supported the view that oxidative stability in these commercial PSOs was more closely associated with antioxidant-related constituents than with major unsaturated fatty acids alone. Total phenolics, total sterols, δ-tocopherol, and total tocopherols showed positive relationships with oxidation induction time, and these variables contributed strongly to PCA-based discrimination among samples. By contrast, visual color did not track reliably with antioxidant constituents or stability-related markers, indicating that appearance should not be used as a proxy for nutritional or oxidative quality. Among the products evaluated, samples 9, 15, and 16 consistently displayed higher antioxidant-related profiles together with stronger stability indices, although such rankings should be interpreted within the constraints of accelerated oxidation testing and the analytical methods applied. Several practical implications follow from these findings. First, retention of δ-tocopherol and sterol-related fractions should be prioritized during processing, as these constituents were repeatedly linked with higher stability and antioxidant-related performance. Second, greater transparency in commercial labeling regarding processing conditions, packaging light barrier, and production date would improve interpretability of quality indices and support evidence-based consumer choice. Finally, future research should incorporate compound-resolved profiling of phenolics and sterols and should evaluate stability under realistic storage conditions, including packaging-dependent light exposure, to better connect compositional markers with shelf-life behavior in the marketplace.

## Figures and Tables

**Table 1:** Brands and characteristics corresponding to samples 1–16.

Sample Number	Brand	Processing Method	Place of Production	Container Properties
1	Yishutang	Cold press	Hebei Province	Transparent glass bottle
2	Dearmine	Cold press	Hebei Province	Transparent glass bottle
3	Sanliangyin	Cold press	Hebei Province	Transparent glass bottle
4	Xinqidian	Cold press	Hebei Province	Transparent glass bottle
5	Dongbeilaoliu	Cold press	Jilin Province	Brown glass bottle
6	Changbaigongfang	Cold press	Jilin Province	Brown glass bottle
7	Oubeinuo	Cold press	Jilin Province	Transparent glass bottle
8	Yunxiancun	Cold press	Shanxi Province	Brown glass bottle
9	Wufenghuiguo	Supercritical extraction	Shanxi Province	Brown glass bottle
10	Grandpa’s Farm	Cold press	France	Transparent glass bottle
11	DAUPHIN PASTOUREAU	Cold press	France	Transparent glass bottle
12	Daoxinyuan	Cold press	Guangxi Province	Brown glass bottle
13	Chunweixiaozi	Cold press	Jiangxi Province	Brown glass bottle
14	Chugu	Cold press	Anhui Province	Brown glass bottle
15	Sanmark	Cold press	Liaoning Province	Brown glass bottle
16	Pujiang	Cold press	Jiangsu Province	Transparent glass bottle

**Table 2:** Physicochemical properties of pumpkin seed oil.

Sample Number	Acid Value /mg KOH/g Oil	Iodine Value /g I_2_/100 g Oil	Peroxide Value /mEq O_2_/kg Oil	L *	a *	b *
1	1.40 ± 0.02 ^e^	111.19 ± 6.42 ^cd^	7.72 ± 0.54 ^de^	89.72 ± 0.15 ^a^	1.45 ± 0.09 ^j^	61.18 ± 0.11 ^k^
2	1.36 ± 0.08 ^e^	116.09 ± 3.76 ^bcd^	6.94 ± 0.77 ^e^	83.29 ± 0.31 ^d^	2.03 ± 0.14 ^i^	62.76 ± 0.26 ^j^
3	1.24 ± 0.02 ^e^	107.70 ± 7.61 ^cd^	4.63 ± 0.08 ^g^	79.44 ± 0.56 ^f^	2.69 ± 0.2 ^h^	68.78 ± 0.63 ^h^
4	0.22 ± 0.02 ^h^	106.64 ± 5.06 ^d^	10.03 ± 0.39 ^bc^	84.92 ± 0.37 ^c^	1.09 ± 0.07 ^k^	40.54 ± 0.54 ^n^
5	1.32 ± 0.02 ^e^	117.10 ± 3.67 ^bc^	10.03 ± 0.46 ^bc^	65.01 ± 0.43 ^h^	10.28 ± 0.13 ^b^	76.39 ± 0.31 ^d^
6	0.86 ± 0.02 ^g^	128.88 ± 2.09 ^a^	9.26 ± 0.23 ^c^	64.46 ± 0.37 ^i^	10.35 ± 0.23 ^b^	78.91 ± 0.76 ^c^
7	1.34 ± 0.04 ^e^	115.72 ± 3.02 ^bcd^	6.94 ± 0.77 ^e^	65.3 ± 0.14 ^gh^	11.58 ± 0.01 ^a^	72.24 ± 0.38 ^e^
8	0.88 ± 0.06 ^fg^	132.05 ± 7.22 ^a^	9.26 ± 0.54 ^c^	66.08 ± 0.59 ^f^	10.06 ± 0.11 ^c^	70.45 ± 0.19 ^g^
9	1.64 ± 0.06 ^d^	132.77 ± 0.94 ^a^	8.49 ± 0.39 ^d^	80.53 ± 0.26 ^e^	0.38 ± 0.18 ^m^	60.39 ± 0.49 ^k^
10	2.70 ± 0.06 ^b^	131.45 ± 7.50 ^a^	11.57 ± 0.46 ^a^	66.04 ± 0.02 ^g^	6.52 ± 0.23 ^f^	71.02 ± 0.41 ^f^
11	4.30 ± 0.06 ^a^	131.51 ± 0.63 ^a^	10.80 ± 0.46 ^ab^	51.51 ± 0.06 ^j^	7.82 ± 0.07 ^e^	80.49 ± 0.52 ^b^
12	1.10 ± 0.02 ^f^	128.16 ± 2.04 ^a^	10.80 ± 0.62 ^ab^	64.43 ± 0.04 ^i^	8.19 ± 0.19 ^d^	83.36 ± 0.44 ^a^
13	2.24 ± 0.02 ^c^	132.49 ± 9.24 ^a^	10.03 ± 0.46 ^bc^	86.05 ± 0.19 ^b^	−0.38 ± 0.04 ^n^	65.43 ± 0.38 ^i^
14	0.32 ± 0.04 ^h^	132.33 ± 4.35 ^a^	10.80 ± 0.77 ^ab^	89.44 ± 0.17 ^a^	−1.76 ± 0.16 ^o^	44.49 ± 0.66 ^m^
15	0.4 ± 0.00 ^h^	123.14 ± 4.51 ^ab^	6.17 ± 0.46 ^f^	83.16 ± 0.26 ^d^	0.63 ± 0.14 ^l^	50.04 ± 0.45 ^l^
16	2.14 ± 0.02 ^c^	132.48 ± 4.17 ^a^	10.80 ± 0.46 ^ab^	65.29 ± 0.51 ^h^	6.21 ± 0.05 ^g^	72.26 ± 0.59 ^e^

* Values are presented as mean ± standard deviation (*n* = 3). Different superscript letters within the same column indicate statistically significant differences (*p* < 0.05).

**Table 3:** Fatty acid composition (%) of pumpkin seed oil.

Sample	C12:0	C16:0	C18:0	C18:1	C18:2	C18:3	C23:0	MUFA	PUFA
1	0.10 ± 0.07 ^b^	13.15 ± 0.06 ^b^	7.55 ± 0.03 ^c^	26.06 ± 0.05 ^g^	52.05 ± 0.05 ^c^	0.88 ± 0.03 ^d^	0.21 ± 0.00 ^c^	26.06 ± 0.05 ^g^	52.93 ± 0.05 ^e^
2	ND	12.97 ± 0.07 ^bc^	7.76 ± 0.02 ^c^	25.62 ± 0.02 ^gh^	52.74 ± 0.02 ^c^	0.91 ± 0.05 ^d^	ND	25.62 ± 0.02 ^gh^	53.66 ± 0.30 ^cd^
3	ND	12.50 ± 0.06 ^cd^	7.02 ± 0.16 ^d^	35.50 ± 0.14 ^a^	44.99 ± 0.01 ^e^	ND	ND	35.50 ± 0.14 ^a^	44.99 ± 0.01 ^j^
4	ND	11.86 ± 0.09 ^e^	7.66 ± 0.04 ^c^	31.04 ± 0.06 ^c^	45.34 ± 0.03 ^e^	4.10 ± 0.04 ^a^	ND	31.04 ± 0.06 ^c^	49.44 ± 0.19 ^gh^
5	ND	13.39 ± 0.07 ^b^	7.93 ± 0.01 ^b^	27.56 ± 0.02 ^ef^	47.08 ± 0.07 ^de^	2.74 ± 0.04 ^b^	1.29 ± 0.03 ^a^	27.56 ± 0.02 ^ef^	49.83 ± 0.08 ^g^
6	0.10 ± 0.07 ^b^	12.00 ± 0.16 ^de^	6.59 ± 0.10 ^e^	27.59 ± 0.24 ^ef^	52.96 ± 0.37 ^ab^	0.77 ± 0.05 ^e^	ND	27.59 ± 0.24 ^ef^	53.73 ± 0.02 ^c^
7	0.13 ± 0.06 ^a^	14.52 ± 0.08 ^a^	9.17 ± 0.13 ^a^	25.13 ± 0.92 ^h^	48.46 ± 0.04 ^d^	0.75 ± 0.04 ^e^	0.63 ± 0.01 ^b^	25.13 ± 0.92 ^h^	49.21 ± 0.04 ^h^
8	ND	8.33 ± 0.10 ^g^	4.74 ± 0.28 ^h^	32.45 ± 0.13 ^b^	54.48 ± 0.04 ^abc^	ND	ND	32.45 ± 0.13 ^b^	54.48 ± 0.05 ^b^
9	0.10 ± 0.07 ^b^	12.57 ± 0.05 ^cd^	7.69 ± 0.11 ^c^	25.78 ± 0.05 ^g^	52.81 ± 0.11 ^c^	0.74 ± 0.02 ^e^	ND	25.78 ± 0.05 ^g^	53.56 ± 0.03 ^cd^
10	ND	12.17 ± 0.06 ^de^	6.96 ± 0.01 ^d^	27.04 ± 0.22 ^f^	52.86 ± 0.05 ^c^	0.98 ± 0.04 ^d^	ND	27.04 ± 0.22 ^f^	53.84 ± 0.03 ^c^
11	ND	12.35 ± 0.02 ^de^	6.48 ± 0.08 ^e^	27.78 ± 0.04 ^e^	52.92 ± 0.01 ^c^	0.47 ± 0.02 ^f^	ND	27.78 ± 0.04 ^e^	53.39 ± 0.05 ^d^
12	ND	11.21 ± 0.04 ^f^	5.75 ± 0.04 ^f^	29.68 ± 0.04 ^d^	52.01 ± 0.05 ^c^	0.68 ± 0.02 ^e^	0.67 ± 0.02 ^b^	29.68 ± 0.04 ^d^	52.69 ± 0.07 ^e^
13	ND	13.27 ± 0.07 ^b^	6.91 ± 0.04 ^d^	25.00 ± 0.13 ^h^	53.86 ± 0.07 ^bc^	0.96 ± 0.03 ^d^	ND	25.00 ± 0.13 ^h^	54.82 ± 0.03 ^b^
14	ND	6.29 ± 0.05 ^h^	5.11 ± 0.06 ^g^	21.79 ± 0.09 ^i^	46.04 ± 0.13 ^de^	1.41 ± 0.01 ^c^	ND	21.79 ± 0.09 ^i^	47.46 ± 0.04 ^i^
15	ND	12.32 ± 0.12 ^de^	2.29 ± 0.01 ^i^	27.13 ± 0.03 ^f^	57.03 ± 0.03 ^a^	1.24 ± 0.03 ^c^	ND	27.13 ± 0.03 ^f^	58.27 ± 0.08 ^a^
16	ND	12.45 ± 0.06 ^cd^	4.92 ± 0.02 ^gh^	30.81 ± 0.11 ^c^	51.82 ± 0.15 ^c^	ND	ND	30.81 ± 0.11 ^c^	51.82 ± 0.03 ^f^

Values are presented as mean ± standard deviation (*n* = 3). Different superscript letters within the same column indicate statistically significant differences (*p* < 0.05). ND: Undetected.

**Table 4:** Correlation analysis of compositional indices with oxidative stability and radical scavenging activity.

Active Ingredients	DPPH Radical Scavenging Rate	ABTS Cation Radical Scavenging Rate	Oxidation Induction Time
Total phenolics	0.46 *	0.626 **	0.9 **
Total sterol	0.41 *	0.551	0.743 **
α-tocopherol	0.286	0.42	0.396
γ-tocopherol	0.226	0.415	0.46
δ-tocopherol	0.427 *	0.461	0.702 **
Total tocopherol	0.427 *	0.482	0.649 **
C18:0	0.117	−0.26	0.09
C18:1	−0.126	−0.06	−0.357
C18:2	0.042	0.225	0.411
Unsaturated fatty acid	0.059	−0.051	0.203

*: significant correlation (*p* < 0.05); **: extremely significant correlation (*p* < 0.01).

## Data Availability

The original contributions presented in the study are included in the article, further inquiries can be directed to the corresponding author.
